# Liquid biopsy based on whole blood transcriptome and artificial intelligence for the prediction of coronary artery calcification: a pilot study

**DOI:** 10.1093/ehjdh/ztaf042

**Published:** 2025-05-02

**Authors:** Rosana Poggio, Gaston A Rodriguez-Granillo, Florencia De Lillo, Alejandra Bibiana Rubilar, Sarah Y Garron-Arias, Nelba Pérez, Razan Hijazi, Claudia Solari, María Olivera-Mores, Soledad Rodriguez-Varela, Alan Möbbs, Estefanía Mancini, Ignacio Berdiñas, Alejandro La Greca, Carlos Luzzani, Santiago Miriuka

**Affiliations:** MultiplAI Health, 184 Cambridge Science Park Rd, Milton, Cambridge CB4 0GA, United Kingdom; Instituto Médico ENERI, Clinica La Sagrada Familia, Av. del Libertador 6647, Cdad, Autónoma de Buenos Aires, Argentina; MultiplAI Health, 184 Cambridge Science Park Rd, Milton, Cambridge CB4 0GA, United Kingdom; Instituto Médico ENERI, Clinica La Sagrada Familia, Av. del Libertador 6647, Cdad, Autónoma de Buenos Aires, Argentina; Instituto Médico ENERI, Clinica La Sagrada Familia, Av. del Libertador 6647, Cdad, Autónoma de Buenos Aires, Argentina; Department of Cardiovascular Imaging, LIAN, Instituto de Neurociencias (INEU), Fleni-CONICET, RN 9 Km 53, Loma Verde, Provincia de Buenos Aires, Argentina; MultiplAI Health, 184 Cambridge Science Park Rd, Milton, Cambridge CB4 0GA, United Kingdom; MultiplAI Health, 184 Cambridge Science Park Rd, Milton, Cambridge CB4 0GA, United Kingdom; MultiplAI Health, 184 Cambridge Science Park Rd, Milton, Cambridge CB4 0GA, United Kingdom; MultiplAI Health, 184 Cambridge Science Park Rd, Milton, Cambridge CB4 0GA, United Kingdom; Department of Cardiovascular Imaging, LIAN, Instituto de Neurociencias (INEU), Fleni-CONICET, RN 9 Km 53, Loma Verde, Provincia de Buenos Aires, Argentina; MultiplAI Health, 184 Cambridge Science Park Rd, Milton, Cambridge CB4 0GA, United Kingdom; MultiplAI Health, 184 Cambridge Science Park Rd, Milton, Cambridge CB4 0GA, United Kingdom; MultiplAI Health, 184 Cambridge Science Park Rd, Milton, Cambridge CB4 0GA, United Kingdom; MultiplAI Health, 184 Cambridge Science Park Rd, Milton, Cambridge CB4 0GA, United Kingdom; Department of Cardiovascular Imaging, LIAN, Instituto de Neurociencias (INEU), Fleni-CONICET, RN 9 Km 53, Loma Verde, Provincia de Buenos Aires, Argentina; MultiplAI Health, 184 Cambridge Science Park Rd, Milton, Cambridge CB4 0GA, United Kingdom; MultiplAI Health, 184 Cambridge Science Park Rd, Milton, Cambridge CB4 0GA, United Kingdom; Department of Cardiovascular Imaging, LIAN, Instituto de Neurociencias (INEU), Fleni-CONICET, RN 9 Km 53, Loma Verde, Provincia de Buenos Aires, Argentina

**Keywords:** Transcriptome, Coronary calcium, Artificial intelligence, Machine learning, Liquid biopsy

## Abstract

**Aims:**

Whole blood RNA expression is modulated in response to signals from tissues, including the vessel wall. The primary objective of this study was to explore the ability of whole blood transcriptomes, analysed using artificial intelligence (AI), to predict coronary artery calcifications (CAC).

**Methods and results:**

A total of 196 subjects [men aged 40–70 years and women aged 50–70 years without known cardiovascular disease (CVD)] were non-consecutively enrolled for CAC assessment via chest computed tomography. Whole blood RNA was isolated and sequenced. Different AI models were trained using clinical and transcriptomic variables as distinctive features to identify the presence of CAC (Agatston score >0). Finally, we compared the predictive performance of these models. The prevalence of CAC was 43.9%. The combined AI model, incorporating transcriptome data along with age, sex, body mass index, smoking status, diabetes, and hypercholesterolaemia, achieved an area under the curve (AUC) of 0.92 (95% CI, 0.88–0.95) for predicting the presence of CAC, with a sensitivity of 92%, specificity of 80%, positive predictive value of 81%, negative predictive value of 91%, and an overall accuracy of 86%. The combined AI model demonstrated significantly improved discrimination compared with the transcriptomic model (AUC 0.79; *P* = 0.009), the clinical variables model (AUC 0.72; *P* < 0.001), and the CVD risk model (AUC 0.68; *P* < 0.001).

**Conclusion:**

In this pilot study, an AI model integrating whole blood transcriptome data with clinical risk factors demonstrated the ability to predict CAC, providing incremental value over clinical models. Further studies are needed to achieve more robust validation.

## Graphical Abstract

**Graphical Abstract:**
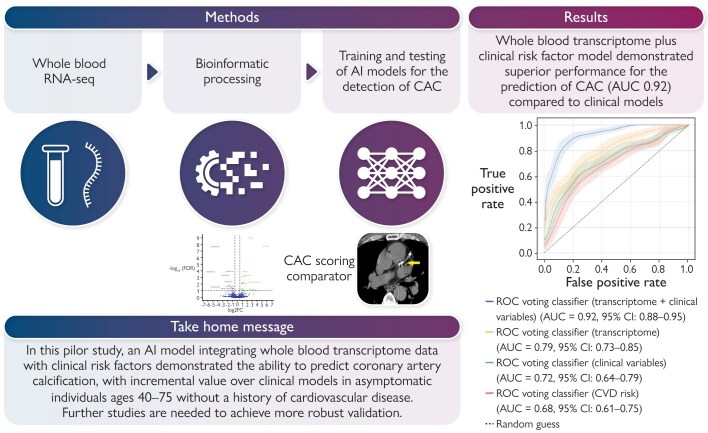

## Background

The concept of a liquid biopsy has gained momentum in recent years due to its potential to detect disease signals in the blood.^1^ This methodology involves the analysis of various blood biomarkers, such as cell-free DNA (cfDNA), RNA, and proteins and has been explored for both disease screening and prognosis.^2^

Among the numerous molecules utilized in omics analyses, RNA stands out due to its central role in biological processes. As a dynamic copy of DNA, RNA expression responds to environmental influences according to inherited patterns. Deep RNA sequencing of peripheral blood may provide extensive insights into various diseases, particularly vascular diseases, as the interaction between blood and the arterial wall generates information that can be detected in the bloodstream. It has been established that alterations in gene expression are associated with the presence of atherosclerosis, coronary artery disease, and stroke.^3^ Despite these advances, most developments in liquid biopsy have focused on cancer detection, with relatively few studies conducted in CVD.^4–6^

Accordingly, this pilot clinical study aimed to explore the ability of the whole blood transcriptome, analysed using artificial intelligence (AI) algorithms, to predict the presence of coronary artery calcification (CAC) as a proxy for coronary atherosclerosis in asymptomatic individuals without a prior history of CVD.

## Methods

### Study population

This study was reviewed and approved by the Institutional Review Ethics Board. An opportunistic sampling method was employed at a healthcare clinic in Argentina to recruit 200 non-consecutive patients (men aged 40–75 years and women aged 50–75 years) who were referred for chest CT evaluation (e.g. due to symptoms such as cough or a history of smoking) or individuals attending the clinic for other reasons who volunteered for coronary artery calcium (CAC) assessment using low-dose chest computed tomography. Eligible participants had no prior history of atherosclerotic cardiovascular disease (ASCVD) and provided informed consent to participate in the study.

Patients were systematically excluded if they had a documented history of chronic kidney or liver failure, exacerbated asthma or chronic obstructive pulmonary disease (COPD), pulmonary fibrosis, recent acute myocardial infarction, heart failure, prior coronary or other vascular interventions, uncontrolled hyper- or hypothyroidism, adrenal insufficiency, recent surgery within the last three months, significant trauma within the last 6 months (defined as involving bone fractures and/or surgical interventions), active or ongoing treatment for known oncological diseases, ongoing pregnancy, puerperium of <12 months postpartum, immunosuppressive treatment, or confirmed COVID-19 within the last 3 months.

### Data collection

Participants who met the eligibility criteria were invited to take part in the study, and informed consent was obtained. Study data were collected using questionnaires specifically designed for this research. The collected data included several parameters: age, sex, clinical history of diabetes, hypertension, smoking habits, and medications at the time of admission. Participants were classified as having hypercholesterolaemia if they had a total cholesterol level >240 mg/dL, LDL cholesterol (LDL-C) >160 mg/dL,^7^ or were documented as being on lipid-lowering medications in their medical records.

Trained and certified personnel collected body weight and height data following standardized procedures using an integrated scale and stadiometer. Blood pressure was measured using a digital blood pressure monitor (Omron, model HEM-7130). Participants were required to remain seated and at rest for 5 min before measurement. The consumption of tea, mate, or coffee, as well as smoking or physical activity, was not permitted within 30 min prior to testing. Three blood pressure measurements were taken at 1-min intervals, and the average of the three readings was used for analysis.^8^

### Blood sample collection

Whole blood (3 mL) was collected via standard venepuncture from the arm into a Tempus tube and stored at −20°C until processing, following the manufacturer’s instructions. Each sample was labelled with a unique identifier number.

### Chest computed tomography scan

A low-dose, ungated chest CT scan was performed using a multidetector spectral tomography system (IQon Spectral CT, Philips Healthcare, The Netherlands) with the following parameters: collimation 64 × 0.625 mm, tube voltage 120 kV, current 70–140 mA based on patient size, rotation time 270 ms, and slice thickness 2.0 mm.

The presence and extent of CAC were assessed using both ordinal variables (number of segments with CAC and number of affected vessels) and continuous variables (Agatston score), employing dedicated software (HeartBeat-CS, Philips Healthcare, Best, The Netherlands). The threshold for CAC detection was defined as a CT attenuation value of 130 HU. A region of interest enclosing these areas was manually drawn, enabling a computer-driven measurement of the calcified lesion area based on the Agatston score. This score was obtained by multiplying each calcified area by a pre-established density factor and summing the individual lesion scores.

On a per-patient basis, the presence of CAC was defined as an Agatston score > 0. Ungated chest CT has demonstrated a high level of agreement with ECG-gated CAC scoring, offering similar prognostic value and reliable discrimination between CAC categories.^9,10^

### RNA sequencing

Total RNA was extracted from the collected blood samples using Thermo Fisher’s RNA spin column kit (Thermo Fisher Scientific, Waltham, MA, USA) following the manufacturer’s protocol. Briefly, the frozen blood samples were thawed on ice, and RNA was isolated using a spin column-based purification method. The extracted RNA was eluted in nuclease-free water and quantified using the RNA Broad Range Qubit Assay (Thermo Fisher Scientific, Waltham, MA, USA). RNA quality was assessed using the RNA Integrity Number (eRIN), measured on an Agilent TapeStation 4150.

Library preparation was carried out using Illumina’s Stranded Total RNA with Ribo-Zero Plus kit (Illumina, San Diego, CA, USA) according to the manufacturer’s instructions. This kit enables the depletion of ribosomal and globin RNA while generating stranded RNA libraries. Briefly, 100 ng of RNA with an eRIN of 7 or higher was subjected to rRNA and globin depletion using the kit, followed by fragmentation and complementary DNA (cDNA) synthesis. Adapters compatible with Illumina sequencing were then ligated to the resulting cDNA fragments, and PCR amplification was performed for library indexing and enrichment. The final libraries were assessed for quality and quantified using an Agilent TapeStation 4150 and Qubit dsDNA Broad Range assay, respectively.

The prepared libraries were sequenced on an Illumina NovaSeq 6000 platform (Illumina, San Diego, CA, USA) using S4 flow cell chemistry. Twenty libraries were pooled together, ensuring an equal amount of DNA from each library, and loaded onto a single flow cell lane. Sequencing was performed using 150 bp paired-end reads, targeting a minimum depth of at least 100 million reads per sample. Base calling and quality scoring were conducted using Illumina Real-Time Analysis (RTA) software.

### Bioinformatics and AI analyses

Raw sequencing reads delivered by the NGS provider were quality checked with FastQC software, and any adapter contamination was removed. Good-quality (>Q30) 150 bp-long paired-end reads were aligned to the reference human genome in two-pass mode against the GRCh38 genome using STAR with mostly standard parameters. Quantification of transcripts was performed using SALMON with the GRCh38 genome/transcriptome. Differential expression analysis was performed using R package edgeR; coding and non-coding genes were considered differentially expressed and retained for further analysis when log2FC group2/group1 ≥ 1 and FDR < 0.1. The Python’s Seaborn library was used to generate the volcano plot.

After collecting and analysing bioinformatic data, four distinct AI models were developed using supervised learning techniques. Each model employs a unique approach, incorporating different variables for its predictions. The first model *(CVD Risk model)* incorporates the 10-year CVD risk, estimated using the World Health Organization (WHO) non laboratory based chart specifically developed for the Southern Latin American population.^11^ For the descriptive and analytical purposes of the current study, WHO risk categories were grouped as follows: low risk <10%, 10% to 19% (intermediate risk), and 20% or higher (high risk). The second model exclusively leverages clinical risk factors data *(Clinical variables model)* using body mass index (BMI), hypertension, current smoking status, age, sex, diabetes, and hypercholesterolaemia (*Table 1*). The third model focuses exclusively on transcriptomics variables *(Transcriptomic model)* using a comprehensive list of features, drawing on previous research findings to ensure our model’s relevance and applicability to the analysis. Finally, the fourth model combines transcriptomics and risk factors data (*Combined model*).

**Table 1:** General characteristics of the study population

	Total (*n* 196)	CAC 0 (*n* 100)	CAC >0 (*n* 96)	*P*-value
Age, mean y (SD)		55.2 (8.1)	60.9 (7.9)	<0.01
40–49 y,%	15.3	21.0	9.4	0.04
50–59 y, %	42.9	52.0	33.3	0.01
≥60 y, %	41.8	27.0	57.3	<0.01
Men (%)	55.6	52.0	59.4	0.37
Women (%)	44.4	48.0	40.6	0.37
Diabetes^a^ (%)	12.2	12	12.5	1
Hypertension^b^ (%)	40.8	29.0	46.9	<0.01
Hipercholesterolaemia^c^ (%)	29.6	23.0	36.5	0.01
Current smoker^d^ (%)	14.8	13	16.7	0.60
Obesity^e^ (%)	38.3	39.0	37.5	0.95
Statins treatment (%)	23.0	15.0	31.3	0.01
CVD risk <10%^f^ (%)	63.8	76.0	51.0	
CVD risk 10%–19% (%)	20.9	13.0	31.3	
CVD risk ≥20% (%)	15.3	11.0	17.7	<0.01

^a^Diabetes was defined based on the presence of drug treatment or a confirmed diagnosis based on medical records.

^b^Hypertension was defined as participants with blood pressure values ≥140/90 mmHg or those under drug treatment.

^c^High cholesterol was defined based on the presence of total cholesterol >240 mg/dL, low-density lipoprotein cholesterol (LDL-C) > 160 mg/dL or under lipid-lowering drugs.

^d^Person who currently smokes tobacco products.

^e^Obesity was defined as a BMI ≥ 30 kg/m².

^f^CVD risk: <10% (low risk), 10%–19% (intermediate risk), ≥20% (high risk). y: years.

To maximize the use of our limited dataset for both training and validation, while ensuring a stable metric for model comparison and hyperparameter optimisation, a Leave-One-Out (LOO) cross-validation strategy was employed. This approach involves iteratively training the model on all but one sample and validating it on the held-out sample, repeating this process for each sample in the dataset.

LOO provides a nearly unbiased estimate of model performance, enabling us to evaluate the stability of our models without reserving a large portion of our data for a separate validation set. This approach is particularly beneficial when optimizing hyperparameters, such as the regularization coefficients in our models.

Furthermore, the consistent performance metric derived from LOO cross-validation serves as a valuable guide in our feature selection process. We employed an iterative feature removal approach to address the curse of dimensionality, particularly relevant in our high-dimensional transcriptomic data. By progressively eliminating less important features based on their impact on the LOO performance metric, we aimed to reduce model complexity while maintaining or improving predictive power.^12^

Additionally, a Features Importance Analysis (FIA) was conducted to identify the most relevant genes and clinical variables influencing the model’s prediction. FIA values were calculated to rank the features that had the greatest impact on the prediction of CAC.^13^

### Model performance and comparisons

Model performance was assessed using traditional metrics, including sensitivity, specificity, accuracy, and positive and negative predictive values. The area under the curve (AUC) was calculated from the receiver operating characteristic curve across different classification thresholds. The AUC was used to compare the prediction performances of different models for the presence of CAC. In order to evaluate the statistical significance between the different models (AUC), we implemented two different approaches. First, DeLong’s test compares the differences between paired AUC values and their standard errors to calculate a *P*-value.^14,15^ Second, a bootstrap hypothesis testing method, using 1000 bootstrap samples, which conducts pairwise comparisons among the models (e.g. clinical vs. transcriptomics, clinical vs. CVD risk) based on the derived *z*-scores and *P*-values (one sided *P*-value).

The optimal threshold, or inflection point, for defining sensitivity and specificity was determined using Youden’s J statistic.^16^ This statistical measure helps identify the threshold that optimizes the overall performance of a binary classification test by balancing sensitivity and specificity.

Net Reclassification Improvement (NRI) was calculated to evaluate the reclassification performance of the CVD risk equation compared to the combined AI model. Participants were categorized into three distinct risk groups: low, intermediate, and high risk.

For participants with CAC > 0 (Cases), upward reclassifications (a higher risk category with the AI model) were identified as correct. Similarly, for individuals with CAC = 0 (Non-cases), downward reclassifications (a lower risk category with the AI model) were considered correct.

The NRI was calculated by comparing the reclassification of individuals between the two models using the R package. The NRI was calculated using the following code^17^:


nri_cases=(cases_up/total_cases)−(cases_down/total_cases)

nri_non_cases=(non_cases_down/total_non_cases)−(non_cases_up/total_non_cases)

NRI=nri_cases+nri_non_cases

The Integrated Discrimination Index (IDI) was also calculated, to describe the improvement in a model’s ability to discriminate between cases (CAC > 0) and non-cases (CAC = 0) when a new predictor or model (Combined) is added to the previous one (CVD risk). Mean Probability for Cases (CAC > 0) was defined as: correctly_predicted_cases/total_cases and Mean Probability for Non-Cases (CAC = 0) as: correctly_predicted_non_cases/total_non_cases. The following codes were used for the IDI calculation^17^:


idi_cases=(mean_cases_new−mean_cases_old)

idi_non_cases=(mean_non_cases_old−mean_non_cases_new)

IDI=idi_cases+idi_non_cases

The calibration of the model was evaluated using the Hosmer–Lemeshow test. A non-significant *P*-value (*P* > 0.05) indicates adequate calibration, while a significant result (*P* ≤ 0.05) suggests a suboptimal calibration.

## Results

A total of 196 patients were included in the final analysis after the exclusion of four samples due to the low quality of the RNA sequencing. The comparative clinical characteristics between cases and controls are outlined in *Table 1*. Among them, 96 patients (49%) had coronary calcium (CAC > 0) and were defined as cases, 59.4% in men and 40.6% in women (*P*=0.072). Statin treatment was present in 23.0% of the study population, with 31.3% of those with CAC >0 and 15.0% of those with CAC = 0 receiving statin therapy (*P* = 0.01).

The mean age of cases was higher (61 vs. 55 years; *P* < 0.01), with a lower proportion of females (41% vs. 48%). Among the ASCVD risk factors, the prevalence of diabetes was 12% in both groups. However, cases had a higher prevalence of hypertension (47% vs. 29%; *P* < 0.01), dyslipidaemia (36% vs. 23%; *P* < 0.01), and use of statins (31% vs. 15%; *P* < 0.01). Additionally, a higher percentage of cases were categorized as moderate risk (37.5% vs. 17%) and high risk (7.3% vs. 1%; *P* < 0.01) based on the WHO risk score.

**Figure 1:**
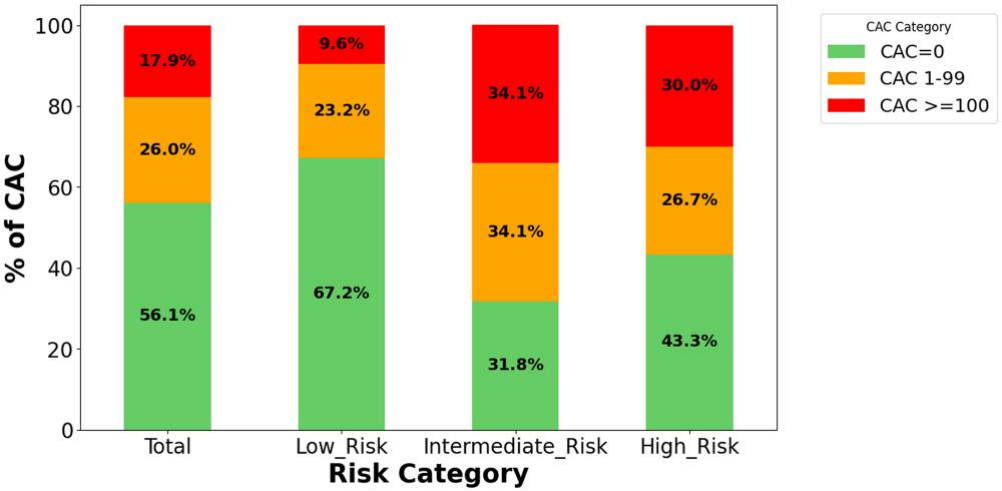
Prevalence of coronary artery calcifications according to cardiovascular risk categories. Prevalence of coronary artery calcifications according to cardiovascular risk categories. The bar plot shows the presence of calcium in the coronary arteries according to the WHO atherosclerotic cardiovascular disease risk and the severity of coronary artery calcifications.

The prevalence of a CAC score > 0 varied across different cardiovascular disease (ASCVD) risk categories. It was found to be 32.8% for individuals classified as low risk, 68.2% for those categorized as intermediate risk, and 56.7% within the high risk category. Similarly, the prevalence of a CAC score equal to or higher than 100AU was 9.6% for those at low/borderline risk, 34.1% for moderate risk, and 52.9% for individuals in the high risk category (*Figure 1*).

### Differential expression analysis for the presence of CAC

The differential expression analysis showed significant alterations in the expression levels of several long non-coding RNAs, including pseudogenes (see Supplementary material online, *Table S1* and Supplementary material online, *Figure S1*). Significant upregulation of SLC8A2 (log2FC 5.397), CREB3L1 (log2FC 2.970), NPIPA9 (log2FC 2.474), RAP1GAP (log2FC 1.496), APOL4 (log2FC 1.249), CD177 (log2FC 1.122), and ARG2 (log2FC 0.959) was observed. By contrast, the mitochondrial-related genes MTND5P11 (log2FC −4.788), MTND4P12 (log2FC −3.897) and MTND4LP30 (log2FC −3.774) were downregulated in individuals with coronary calcifications.

### Key features used by combined model for the prediction of CAC

Different models were built to predict the presence of any CAC. The Features Importance Analysis revealed a diverse set of genes and clinical variables utilized by the combined AI model to predict the presence of CAC. The highest-ranked features, based on feature importance, included JUN (127.0), Age (102.8), GZMK (86.9), IPO9 (86.5), NACC1 (84.2), PLXNC1 (81.6), ACAD10 (81.1), ADPRHL1 (80.5), and CTSF (80.3). Additional details regarding the remaining features used for prediction are provided in Supplementary material online, *Table S2*.

The combined model, which included transcriptomic and clinical variables data, had a sensitivity of 92%, specificity of 80%, positive predictive value of 81%, negative predictive value of 91%, and an overall accuracy of 86%. The false positive rate was 20%, and the false negative rate was 8.33%. There was no clear association between these false results and the clinical variables analysed (see Supplementary material online, *Table S3*).

**Figure 2:**
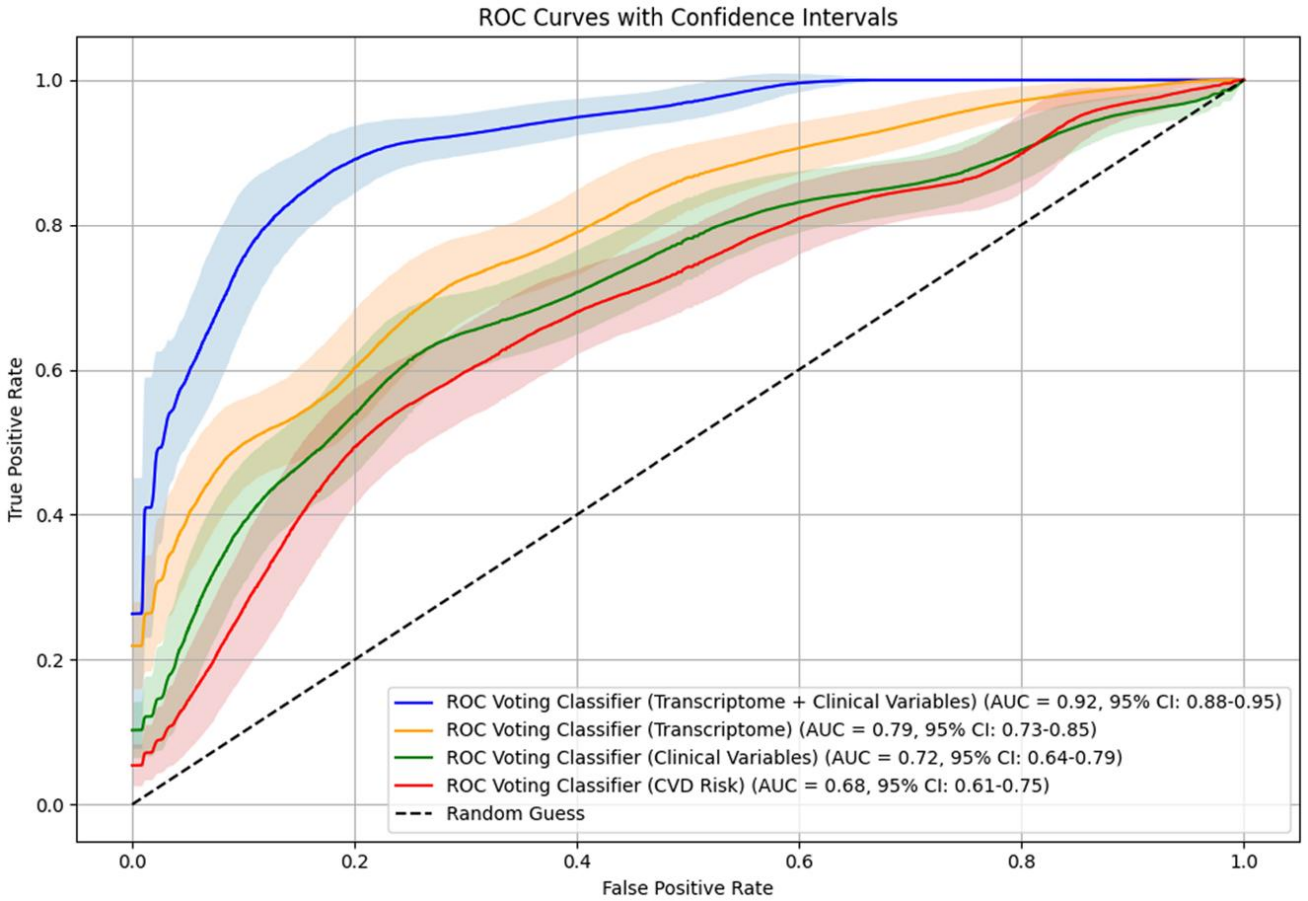
Comparative evaluation of model discrimination for predicting coronary artery calcifications.

The combined model demonstrated superior performance in classifying individuals with CAC > 0, with an AUC of 0.92 (95% CI 0.88–0.95). This was significantly better than the transcriptomic model (AUC 0.79, 95% CI 0.73–0.85; *P* = 0.009), the clinical variables model (AUC 0.71, 95% CI 0.64–0.79; *P* < 0.001), and the CVD risk model (AUC 0.68, 95% CI 0.61–0.76; *P* < 0.001; *Figure 2*).

When the classification performance across different subgroups was explored, the combined model correctly classified 97% of cases (35/36) among participants with CAC >100, 96% (25/26) in those with CAC >0 in three vessels, and 97% (30/31) in those with CAC >0 in the left main coronary artery.

### Net reclassification and integrated discrimination analysis

The data used for the calculation of the NRI is depicted in Supplementary material online, *Table S4*. The NRI for Cases (CAC > 0) was 0.5833. This value indicates that the AI model improved the classification of Cases by 58.33% compared to the CVD-risk model.

The NRI for Non-Cases (CAC = 0) was −0.38. This negative value suggests that the AI model was less effective in correctly reclassifying individuals without CAC. Specifically, it indicates a 38% decrease in correct classification for non-cases, meaning some individuals were incorrectly moved to a higher risk category.

The total NRI of 20.33% reflects the overall improvement in classification accuracy when using the AI model over the CVD-risk model, despite the decrease in accuracy for non-cases.

The IDI analysis showed that the mean predicted probability for correctly classified cases (CAC > 0) was 1.038, while for correctly classified non-cases (CAC = 0), it was 0.856. The overall IDI was 0.138, indicating a 13.8% improvement in the combined model’s ability to distinguish between cases and non-cases compared to the CVD risk model. The Hosmer–Lemeshow test yielded a *P*-value < 0.001, suggesting suboptimal model calibration.

## Discussion

In this pilot clinical study comprising asymptomatic individuals aged 40–75 without a history of CVD, an AI model integrating whole blood transcriptome data with clinical risk factors demonstrated the ability to predict the presence of CAC, with incremental value over clinical models.

Despite a net reclassification improvement of 20%, challenges remain in addressing the overestimation of CAC risk in non-cases, leading to false positives. These misclassifications were not associated with any of the analysed variables, suggesting that unmeasured factors—such as inflammatory processes or alternative forms of subclinical atherosclerosis—may contribute to the discrepancies^18^ (see Supplementary material online, *Table S3*). Supporting this hypothesis, significant alterations were observed in the expression levels of several genes implicated in key mechanisms of atherosclerosis, including endothelial dysfunction, inflammation, and lipid metabolism (see Supplementary material online, *Tables S1* and *S2*). This finding suggests that the model may be capturing relevant molecular signatures associated with CAC development.

The combined model also demonstrated a 14% higher discrimination capacity between cases and non-cases compared to the baseline CVD risk model; however, further refinement is required due to its suboptimal calibration.

In a single study, a plasma microRNA panel was used to predict the presence of CAC in patients diagnosed with rheumatoid arthritis. The improvement in prediction accuracy was relatively modest when compared solely to clinical factors (c-statistic net difference 0.01 for total cases and 0.05 for severe CAC).^19^

In the PREDICT study, a genetic expression score (GES) was developed using a panel of 23 genes to predict obstructive coronary artery disease (CAD, defined as ≥50% stenosis) in symptomatic, non-diabetic individuals referred for invasive coronary angiography. This score was derived from a blood-based gene expression (RNA) panel, previously selected through microarray analysis. While the GES demonstrated promising sensitivity (83%), it exhibited relatively low specificity (43%).^4^

Similarly, the COMPASS study validated the diagnostic accuracy of the GES for identifying obstructive CAD in and independent symptomatic nondiabetic patients referred for myocardial perfusion imaging, extending the findings from the PREDICT study to a lower-risk population. The GES demonstrated strong discrimination for obstructive CAD, with an AUC of 0.79 (*P* < 0.001), outperforming clinical models. Sensitivity, specificity, and negative predictive value were reported at 89%, 52%, and 96%, respectively. However, despite these favourable outcomes, particularly the high sensitivity and reproducibility, the relatively low specificity implies a potential for increased false positive rates.^5^

Zhang *et al*. utilised RNA sequencing (RNA-seq) to explore differentially expressed genes among individuals with a history of early myocardial infarction (MI), those with high CAC without prior MI, and controls without elevated CAC or MI. The study identified three coding genes (APOD, CLNK, RASGEF1A) and one long intergenic non-coding RNA (lincRNA) (RP11-245J9.5) that were differentially expressed in individuals with high CAC compared to controls. Notably, APOD was significantly downregulated in the high CAC group (FDR = 0.01).^6^

Upon comparing the gene sets from our analysis with those identified in the mentioned studies, no exact gene matches were found. The lack of common genes may reflect variations in study design, population characteristics, gene expression analysis techniques, and the complexity of gene networks involved in atherosclerosis. This underscores the value of a comprehensive transcriptomic approach in capturing a broader range of potential biomarkers.

The predictive capacity of the methodology applied in this study can be attributed to the comprehensive data provided by deep RNA sequencing, the bioinformatic analysis methods, and the machine learning models employed. Over the past decade, RNA expression analysis has garnered increasing attention, as it captures not only genetic predispositions but also the dynamic influence of environmental factors on biological processes. Notably, alterations in gene expression have been associated with the development of atherosclerosis, coronary artery disease, and stroke, underscoring the potential of RNA analysis for CVD prediction.^20^

In addition, this methodology, unlike previous studies, focused on a comprehensive analysis of the entire blood transcriptome rather than a limited gene panel. It incorporated non-coding RNAs, circular RNAs, and isoforms, highlighting the interconnected nature of genes, which may enhance precision in disease prediction.

Although these findings provide a preliminary proof of concept for the use of RNA sequencing in coronary atherosclerosis prevention, the higher initial implementation costs of RNA sequencing and AI-driven models compared to standard CAC screening methods may constrain their widespread applicability as a screening tool. Nonetheless, the potential prognostic value of this methodology in predicting incident cardiovascular events represents a promising area for future research.

This study has several limitations that could potentially impact the results. First, the observed gene expression pattern among cases does not correspond to a specific CAC level but rather reflects molecular alterations associated with an increased likelihood of a CAC score greater than 0. Second, there is a potential for misclassification bias in calcium scoring due to the use of non-gated CT. However, this bias is unlikely to significantly affect the results, given the well-documented high agreement between gated and non-gated CT scans.^9,10^ Notwithstanding, it should be stressed that as an exploratory study, we decided to perform ungated chest CT scans to simultaneously assess the thoracic aortic calcium and the liver fat content, which will be reported independently. Third, the lack of direct laboratory measures in this study may contribute to the inferior performance of the CVD risk model. To address this limitation in the clinical model, we incorporated the diagnosis of hypercholesterolaemia (total or LDL) or the status of being on lipid-lowering treatment from medical records. For the CVD risk assessment, we employed the WHO non-laboratory-based chart specifically developed for the Southern Latin American population. Notably, the predictive performance for CAC in our study (AUC 0.68) closely aligns with that reported by other cholesterol-based risk equations (AUC 0.67–0.73).^21^ Fourth, therapies such as statins and anticoagulants have been associated with CAC progression. However, this effect is unlikely to significantly impact the model’s ability to predict the presence of CAC.^22,23^

Finally, our findings may not be directly extrapolated to other populations, as differences in genomic backgrounds could influence the results. However, we expect the impact of genetic diversity on model performance to be minimal, as gene expression has been extensively demonstrated to be robust across various experimental settings. Additionally, several factors limit the generalizability of our findings, including the opportunistic sampling method, relatively small sample size, extensive exclusion criteria, and suboptimal calibration.

While this study offers a preliminary proof of concept for the use of RNA sequencing in coronary atherosclerosis prevention, these findings should be interpreted with caution due to the study’s limitations. Nonetheless, future studies with larger sample sizes, comprehensive laboratory testing, and clinical outcomes are required to achieve more robust validation.

## Conclusions

In this pilot study, an AI model integrating whole blood transcriptome data with clinical risk factors demonstrated the ability to predict CAC, providing incremental value over clinical models in asymptomatic individuals aged 40–75 without a history of CVD. Further studies are needed to achieve more robust validation.

## Lead author biography


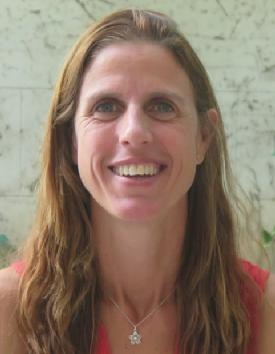


Dr. Rosana Poggio, a cardiologist with an MSc and PhD in health science, is a senior researcher at the National Research Council in Argentina and a senior scholar at Harvard T.H. Chan. With 15 years of experience in designing and implementing clinical studies and cluster trials focused on cardiovascular disease prevention, she has also worked at the Ministry of Health in Argentina. Currently, as the Head of Medical Affairs at MultiplAI Health Ltd., she has designed and conducted three clinical studies to evaluate the accuracy of whole blood RNA-Seq in predicting different stages of subclinical atherosclerosis.

## Supplementary Material

ztaf042_Supplementary_Data

## Data Availability

Raw data is available upon request for academic use under appropriate data-sharing agreements.
