# Supplementary material for: Liquid biopsy based on whole blood transcriptome and artificial intelligence for the prediction of coronary artery calcification: a pilot study
Source: Eur Heart J Digit Health. 2025 May 2;6(4):587–94. doi: 10.1093/ehjdh/ztaf042 (PMC12282340; doi:10.1093/ehjdh/ztaf042)
Supplement: ztaf042_Supplementary_Data [file ztaf042_supplementary_data.zip › Supplementary Material-Feb 2025.docx]

Supplementary Tables and Figures

**Supplementary Figure 1. Volcano plot showing differentially expressed genes based on the presence of coronary calcium score >0.**

(Supplementary Figure 1 uploaded as a single file)

Log2FC: Log 2 fold change. FDR: False Discovery Rate.

**Supplementary Table 1. Genes found differentially expressed between those subjects with or without calcification in the coronary arteries.**

| **Gene Symbol** | **Gene type** | **Gene Name** | **log2Fold Change** | **p-value** | **FDR** | **Function** | **Possible relation to CAC** |
| --- | --- | --- | --- | --- | --- | --- | --- |
| SLC8A2 | Protein Coding | Solute Carrier Family 8 Member A2 | 5.39729127458581 | 7.26909448817555E-13 | 1.72412017617792E-08 | The SLC8A2 gene encodes the protein Na+/Ca2+ exchanger 2 (NCX2), a member of the sodium/calcium exchanger family. These proteins play a critical role in regulating intracellular calcium levels. | Previously related to ischemia^1^ |
| CREB3L1 | Protein Coding | CAMP Responsive Element Binding Protein 3 Like 1 | 2.97033654156435 | 5.16969961426461E-05 | 0.0817450135339568 | Also known as Luman or LZIP, is a transcription factor that belongs to the CREB/ATF (activating transcription factor) family. It is an endoplasmic reticulum (ER) membrane-bound protein involved in various cellular processes, including transcriptional regulation, response to ER stress, cell differentiation and development, and Lipid metabolism | Accelerates vascular calcifications^2^ |
| NPIPA9 | Protein Coding | Nuclear Pore Complex Interacting Protein Family, Member A9 | 2.47484266097469 | 3.51179800802629E-05 | 0.0574445386574977 | Nuclear pore complexes (NPC) are large protein assemblies that bridge the double membrane of the nuclear envelope, which separates the nucleus from the cytoplasm in eukaryotic cells. The NPC functions as a gatekeeper, regulating the transport of molecules to and from the nucleus. Proteins interacting with the NPC are crucial for various cellular processes, including nucleocytoplasmic transport, NPC assembly, cell signaling and homeostasis, and gene expression | None |
| ERFE | Protein Coding | Erythroferrone | 2.47178812567606 | 2.08134441732208E-14 | 9.87327351245076E-10 | Erythroferrone (ERFE) is a hormone produced by erythroblasts (immature red blood cells) in the bone marrow in response to erythropoietin (EPO), which is the hormone that stimulates red blood cell production (erythropoiesis). ERFE plays a significant role in iron homeostasis, particularly during conditions of increased erythropoiesis, such as anaemia or hypoxia (low oxygen levels). The primary function of erythroferrone is to modulate the activity of hepcidin, increase in Iron Availability, and support for Erythropoiesis | Related to cardiovascular events in chronic kidney disease^3^ and metabolic syndrome in a rat model^4^ |
| LOC107986954 | lncRNA | None | 2.47073470671437 | 1.21518046555781E-06 | 0.00480370964538881 | Uncharacterised | None |
| RAP1GAP | Protein Coding | RAP1 GTPase Activating Protein | 1.4964423720055 | 7.46070377419263E-08 | 0.00044239175617047 | RAP1 GTPase Activating Protein (RAP1GAP) is a protein that regulates the function of a small GTPase called RAP1. GTPases are a family of hydrolase enzymes that can bind and hydrolyse guanosine triphosphate (GTP). RAP1 is involved in various cellular processes, including cell adhesion, cell junction formation, and integrin-mediated signalling, which are important for maintaining cellular structure and signalling pathways. | Involved in atherosclerotic aortic calcification^5^ |
| ARL14EP-DT | lncRNA | ARL14EP Divergent Transcript | 1.35912359158555 | 6.77432305775019E-05 | 0.0918153036829987 | A long non-coding transcriptionally associated with ARL14EP, also known as immune-associated nucleotide-binding protein 1 (IAN1), is a member of the ARL (ADP-ribosylation factor-like) family of proteins. The ARL family is a group of small GTPases that are involved in a variety of cellular processes including membrane trafficking, cytoskeletal dynamics, and intracellular signalling. | None |
| LINC01218 | lncRNA | EMCN Intronic Transcript 1 | 1.33288949168988 | 1.14841546816203E-06 | 0.00480370964538881 | Uncharacterized as lncRNA, but EMCN, also known as Endomucin, is a mucin-like sialoglycoprotein that is predominantly expressed on the surface of endothelial cells, particularly within the vascular endothelium. It plays a role as a component of the endothelial glycocalyx, a layer of membrane-bound macromolecules that lines the luminal surface of blood vessels. The specific functions of endomucin include vascular homeostasis, regulation of Cell Adhesion, blood vessel Barrier Function, angiogenesis | No relationship found for LINC01218, but EMCN has been previously linked to CAD in a GWAS study^6^ |
| APOL4 | Protein Coding | Apolipoprotein L4 | 1.24930831555304 | 1.02909439238266E-08 | 0.000110747898564507 | Apolipoprotein L4 (ApoL4) is a member of the apolipoprotein L (apoL) family, which is part of a larger group of proteins known as the apolipoproteins. Apolipoproteins are involved in lipid metabolism and are the protein components of lipoproteins, which are particles responsible for transporting lipids, such as cholesterol and triglycerides, through the bloodstream. The apolipoprotein L family members are known to have diverse functions, some of which include roles in lipid transport, programmed cell death (apoptosis), and potentially innate immunity. | None |
| ENSG00000253197 | lncRNA | None | 1.22802505369653 | 5.88012350265095E-05 | 0.0860585984575619 | Uncharacterised | None |
| TMEM212 | Protein Coding | Transmembrane Protein 212 | 1.13569860440697 | 3.01697485310024E-05 | 0.0539919227958382 | TMEM212 is a protein that, as its name implies, contains a region that spans the membrane of a cell. Transmembrane proteins are integral to a wide range of biological functions, typically serving as channels, transporters, receptors, or enzymes involved in various cellular processes. However, detailed information about the specific function of TMEM212 is not available. | None |
| CD177 | Protein Coding | Human Neutrophil Alloantigen 2a | 1.12261578574222 | 6.1849192901665E-05 | 0.0862923577551848 | CD177 is primarily known for its role in neutrophil biology and the immune response. CD177's functions include Transendothelial Migration, Vascular Inflammation, Involvement in Vascular Diseases, and Vascular Endothelial Interaction. | Involved in many vasculitis, including Kawasaki Disease^7^ and in coronary artery disease^8^ |
| THEM5 | Protein Coding | Thioesterase superfamily member 5 | 1.02839047297043 | 2.20774557778398E-06 | 0.00748063049809562 | THEM5 is a protein in the thioesterase enzyme family, which includes enzymes that cleave thioester bonds in various substrates. THEM5 was identified relatively recently, so compared to many other proteins and enzymes, less is known about its specific biological functions. Some possible functions include ipid metabolism and apoptosis pathways and Regulation of Eicosanoid Production, such as prostaglandins and leukotrienes. | None |
| ARG2 | Protein Coding | Arginase 2 | 0.959109858682589 | 1.11412694299548E-07 | 0.000587231553276409 | Arginase 2 (ARG2) is an enzyme that catalyses the hydrolysis of arginine to ornithine and urea. The functional roles of ARG2 include Urea Cycle, Polyamine Synthesis, Nitric Oxide Regulation, Tissue Remodelling and Healing, Cell Proliferation and Immunity, and Metabolic Regulation. Alterations in ARG2 expression and activity have been associated with various pathological conditions, including cardiovascular diseases, inflammatory conditions, certain cancers, and metabolic disorders. As such, the arginase pathway, including ARG2, is a target of interest for therapeutic intervention in these diseases. | Involved in atherosclerosis^9^ and metabolic diseases^10^ |
| EEF1A1P4 | Processed Pseudogene | Eukaryotic Translation Elongation Factor 1 Alpha 1 Pseudogene 4 | -1.05554357664594 | 3.07309044730407E-05 | 0.0539919227958382 | Pseudogene of EEF1A1 | None |
| VANGL2 | Protein Coding | ANGL Planar Cell Polarity Protein 2 | -1.11785175583377 | 3.31692352710932E-05 | 0.0561946076269589 | VANGL2 is a core component of the planar cell polarity (PCP) pathway, which is a conserved molecular signalling cascade that regulates the orientation and coordinated arrangement of cells within the plane of a tissue, orthogonal to the apical-basal axis. PCP signalling is crucial for various processes during embryonic development and in adult tissue homeostasis. | None |
| MTCO3P12 | Unprocessed Pseudogene | MT-CO3 Pseudogene 12 | -1.148600218141 | 8.55057721042123E-07 | 0.00405613731130751 | A pseudogene of MT-CO3, which stands for mitochondrial cytochrome c oxidase subunit III, which is an essential protein component of the cytochrome c oxidase complex (also known as Complex IV) involved in the mitochondrial electron transport chain. | None |
| IGKV2D-30 | IG_V_gene | Immunoglobulin | -1.28354837031931 | 1.18992874473149E-05 | 0.029708763086225 | IGKV2D-30 gene is involved in encoding the kappa type light chain of immunoglobulins. | None |
| MTCO1P40 | Processed Pseudogene | MT-CO1 Pseudogene 40 | -1.63416239427462 | 4.63239300101596E-06 | 0.0137341766743246 | MTCO1P40 is a pseudogene of MTCO1, which encodes the cytochrome c oxidase subunit I, which is a core component of Complex IV of the mitochondrial respiratory chain. This complex is crucial for the final step of the electron transport chain, where it catalyses the transfer of electrons to oxygen, reducing it to water. This process is coupled with the pumping of protons from the mitochondrial matrix to the intermembrane space, helping to maintain the proton gradient necessary for the synthesis of ATP by ATP synthase. | MTCO1P40 has not been directly link to CAD, but its parenteral gene has been implicated suggested as a potential marker of CVD^11^, and also implicated in CAD^12^ |
| MTCO1P12 | Unprocessed Pseudogene | MT-CO1 Pseudogene 12 | -1.83678999267187 | 7.23473592883347E-08 | 0.00044239175617047 | MTCO1P12 is a pseudogene of MTCO1. | Same as before. |
| ITIH5 | Protein Coding | Inter-alpha-trypsin inhibitor heavy chain 5 | -2.58849782937774 | 1.94022586073861E-05 | 0.0438278543599322 | The ITIH5 gene encodes a protein known as inter-alpha-trypsin inhibitor heavy chain 5, which is a member of the inter-alpha-trypsin inhibitor (ITI) family. Proteins from the ITI family are involved in the stabilization of the extracellular matrix (ECM) and play roles in various physiological and pathological processes, including inflammation, cancer, and tissue remodelling. | It has been linked to epigenetic alterations associated with BMI^13^. |
| MTND4LP30 | Processed Pseudogene | MT-ND4L Pseudogene 30 | -3.77469130617047 | 9.80079125999918E-06 | 0.0258288963889212 | MTND4LP30 is a pseudogene of MTND4L | None |
| MTND4P12 | Processed Pseudogene | MT-ND4 Pseudogene 12 | -3.89727783874603 | 1.33699193427133E-12 | 2.11409621286764E-08 | MTND4P12 is a pseudogene of MTND4 | None |
| MTND5P11 | Processed Pseudogene | MT-ND5P Pseudogene 11 | -4.78817011721392 | 1.6307615673182E-08 | 0.000128930727448122 | MTND5P11 is a pseudogene of MTND5 | None |

**Supplementary Table 2. Key Features and Clinical Variables Influencing Prediction in the Combined AI Model.**

| **Gene Id** | **Gene symbol** | **Ranking by feature importance** |
| --- | --- | --- |
| ENSG00000177606 | JUN | 127.03800558154700 |
| Age | Age | 102.81027037349100 |
| ENSG00000113088 | GZMK | 86.97716255705210 |
| ENSG00000198700 | IPO9 | 86.52858753410000 |
| ENSG00000267512 | NACC1 | 84.23266180634310 |
| ENSG00000136040 | PLXNC1 | 81.6052903970711 |
| ENSG00000111271 | ACAD10 | 81.1075923462491 |
| ENSG00000153531 | ADPRHL1 | 80.5562451652912 |
| ENSG00000174080 | CTSF | 80.37649105290730 |
| ENSG00000279235 | Uncategorized (Tec) | 79.06188120702330 |
| ENSG00000276557 | TRBV18 | 76.92504934120670 |
| ENSG00000237638 | LINC02245 | 75.5960225845363 |
| ENSG00000113761 | ZNF346 | 74.61901169362470 |
| ENSG00000137714 | FDX1 | 69.86053590499240 |
| ENSG00000173258 | ZNF483 | 67.46061175165100 |
| ENSG00000102755 | FLT1 | 64.893598649956 |
| ENSG00000163596 | ICA1L | 64.66419193189960 |
| ENSG00000198324 | PHETA1 | 63.88714547043580 |
| ENSG00000122965 | RBM19 | 60.78026594656650 |
| ENSG00000132256 | TRIM5 | 59.88421068832990 |
| ENSG00000115486 | GGCX | 56.89351633498500 |
| ENSG00000276819 | TRBV15 | 55.96239904750370 |
| ENSG00000204469 | PRRC2A | 54.44997705981240 |
| ENSG00000126821 | SGPP1 | 54.20721169571780 |
| ENSG00000197128 | ZNF772 | 53.400252355352000 |
| ENSG00000179820 | MYADM | 53.39103102231310 |
| ENSG00000198208 | RPS6KL1 | 53.3285065234599 |
| ENSG00000006327 | TNFRSF12A | 53.24905234174940 |
| ENSG00000140932 | CMTM2 | 53.086019874024900 |
| ENSG00000161395 | PGAP3 | 53.06409861849120 |
| ENSG00000011028 | MRC2 | 52.27645127493540 |
| ENSG00000012232 | EXTL3 | 52.14884224583580 |
| ENSG00000153774 | CFDP1 | 51.94706820210600 |
| Sex | Sex | 51.65424029830480 |
| ENSG00000064989 | CALCRL | 51.33688391282120 |
| ENSG00000109107 | ALDOC | 50.616395082318400 |
| ENSG00000185697 | MYBL1 | 49.190827633859900 |
| ENSG00000115170 | ACVR1 | 48.63774762331760 |
| ENSG00000283050 | GTF2IP12 | 48.45422617443610 |
| ENSG00000134222 | PSRC1 | 47.81196343514100 |
| ENSG00000110076 | NRXN2 | 47.068482761356300 |
| ENSG00000166387 | PPFIBP2 | 46.97069751327650 |
| ENSG00000261485 | PAN3-AS1 | 46.9538314499973 |
| ENSG00000235290 | HLA-W | 46.77252373013950 |
| ENSG00000168477 | TNXB | 45.36155104262960 |
| ENSG00000182511 | FES | 44.769426621521300 |
| ENSG00000252438 | None | 44.74165139166250 |
| ENSG00000204528 | PSORS1C3 | 44.5551000861233 |
| ENSG00000166822 | TMEM170A | 44.136341991011200 |
| ENSG00000183386 | FHL3 | 43.96339330243040 |
| ENSG00000131127 | ZNF141 | 43.24691045243520 |
| ENSG00000132823 | OSER1 | 43.19463074407270 |
| ENSG00000142867 | BCL10 | 43.09614704776560 |
| ENSG00000173588 | CEP83 | 42.69608241379510 |
| ENSG00000243927 | MRPS6 | 42.31170730371190 |
| ENSG00000007168 | PAFAH1B1 | 42.20641227328900 |
| ENSG00000178913 | TAF7 | 42.19346872163430 |
| ENSG00000085117 | CD82 | 42.09264835503700 |
| ENSG00000023902 | PLEKHO1 | 41.70773998365560 |
| ENSG00000138623 | SEMA7A | 40.71140476981390 |
| ENSG00000151693 | ASAP2 | 40.412971058940500 |
| ENSG00000102978 | POLR2C | 40.33309649528650 |
| ENSG00000124181 | PLCG1 | 38.66510691748780 |
| ENSG00000142046 | TMEM91 | 38.28898476141700 |
| ENSG00000135114 | OASL | 37.97601337495220 |
| ENSG00000082269 | FAM135A | 37.942180594379200 |
| ENSG00000131149 | GSE1 | 37.43320973173000 |
| ENSG00000070526 | ST6GALNAC1 | 37.29547768253370 |
| ENSG00000286177 | LOC105372401 | 37.04442457103800 |
| ENSG00000139626 | ITGB7 | 36.87189645621610 |
| ENSG00000282024 | None | 36.84162071644920 |
| ENSG00000176171 | BNIP3 | 36.46347862298390 |
| ENSG00000150045 | KLRF1 | 36.46270676171610 |
| ENSG00000095139 | ARCN1 | 36.40406289868210 |
| ENSG00000124780 | KCNK17 | 35.9418578632373 |
| ENSG00000249602 | None | 35.83926217405350 |
| ENSG00000088930 | XRN2 | 35.52682146149790 |
| ENSG00000213722 | DDAH2 | 35.474731066910200 |
| ENSG00000211513 | MIR320E | 35.07541690471180 |
| ENSG00000119185 | ITGB1BP1 | 34.57032269981260 |
| ENSG00000005812 | FBXL3 | 34.48420517910490 |
| ENSG00000174106 | LEMD3 | 33.63282406777600 |
| ENSG00000211734 | TRBV5-1 | 33.48450629042860 |
| ENSG00000278740 | None | 33.4532675277153 |
| ENSG00000129355 | CDKN2D | 33.08544530843920 |
| ENSG00000152229 | PSTPIP2 | 32.88645899104280 |
| ENSG00000115415 | STAT1 | 32.38809794522800 |
| ENSG00000218052 | ADAMTS7P4 | 32.266234173581500 |
| ENSG00000166747 | AP1G1 | 32.25792997771320 |
| ENSG00000218283 | MORF4L1P1 | 32.246233509298800 |
| ENSG00000153208 | MERTK | 31.51050062666330 |
| ENSG00000104081 | BMF | 31.11618451064920 |
| ENSG00000066185 | ZMYND12 | 30.869038381711400 |
| ENSG00000259207 | ITGB3 | 30.37194418833450 |
| ENSG00000147889 | CDKN2A | 30.33969652882110 |
| ENSG00000102908 | NFAT5 | 30.284548115022000 |
| ENSG00000105556 | MIER2 | 29.9935247592744 |
| ENSG00000130881 | LRP3 | 29.8769422004768 |
| ENSG00000134242 | PTPN22 | 29.727255924162600 |
| ENSG00000135414 | GDF11 | 29.70072469001190 |
| ENSG00000185745 | IFIT1 | 29.420678077430000 |
| ENSG00000168538 | TRAPPC11 | 29.400526657117900 |
| ENSG00000244754 | N4BP2L2 | 29.293583090308600 |
| ENSG00000087301 | TXNDC16 | 28.97698014684560 |
| ENSG00000177125 | ZBTB34 | 28.78901525497070 |
| ENSG00000204435 | CSNK2B | 28.5012007735616 |
| ENSG00000138246 | DNAJC13 | 28.47063920714480 |
| ENSG00000125388 | GRK4 | 28.27307658861280 |
| ENSG00000154305 | MIA3 | 28.24928796937010 |
| ENSG00000130733 | YIPF2 | 28.2423953711102 |
| ENSG00000143156 | NME7 | 28.20335760107650 |
| ENSG00000140740 | UQCRC2 | 28.15919664612680 |
| ENSG00000117724 | CENPF | 28.072681797092200 |
| ENSG00000175445 | LPL | 27.393704857297500 |
| ENSG00000008869 | HEATR5B | 27.18232615219140 |
| ENSG00000272750 | None | 26.69964094881490 |
| ENSG00000175003 | SLC22A1 | 26.56092029651080 |
| ENSG00000174306 | ZHX3 | 26.101856957155600 |
| ENSG00000163508 | EOMES | 25.61364142231730 |
| ENSG00000150991 | UBC | 25.289402173727400 |
| ENSG00000272325 | NUDT3 | 24.57218944846260 |
| ENSG00000157654 | PALM2AKAP2 | 24.256551411601400 |
| ENSG00000143387 | CTSK | 24.194576864790000 |
| ENSG00000141252 | VPS53 | 23.909449437940600 |
| ENSG00000100603 | SNW1 | 23.49333331913530 |
| ENSG00000143367 | TUFT1 | 23.286047723070500 |
| ENSG00000125510 | OPRL1 | 23.238834553344000 |
| ENSG00000114737 | CISH | 23.16084884926100 |
| ENSG00000142453 | CARM1 | 23.15674441073780 |
| ENSG00000172936 | MYD88 | 22.670900868413500 |
| ENSG00000116030 | SUMO1 | 22.391477595923300 |
| ENSG00000106537 | TSPAN13 | 22.281257161453600 |
| ENSG00000100258 | LMF2 | 22.263951872176300 |
| ENSG00000143452 | HORMAD1 | 21.841008170688700 |
| ENSG00000116016 | EPAS1 | 21.64355212394760 |
| ENSG00000163626 | COX18 | 21.63786378546990 |
| ENSG00000065060 | BLTP3A | 21.541079622293600 |
| ENSG00000196821 | ILRUN | 21.496123338434700 |
| ENSG00000136770 | DNAJC1 | 21.4279679987004 |
| ENSG00000269800 | PLEKHA3P1 | 21.299921088110200 |
| ENSG00000138035 | PNPT1 | 21.13856313372500 |
| ENSG00000099889 | ARVCF | 20.66158058549730 |
| ENSG00000111275 | ALDH2 | 20.63550144245840 |
| ENSG00000038210 | PI4K2B | 20.57126294319790 |
| ENSG00000197467 | COL13A1 | 20.360869068522500 |
| ENSG00000166394 | CYB5R2 | 20.014504813372500 |
| ENSG00000261371 | PECAM1 | 19.718179813427200 |
| ENSG00000134321 | RSAD2 | 19.535368966470100 |
| ENSG00000256553 | TRAV1-2 | 19.29843377084940 |
| ENSG00000085563 | ABCB1 | 18.85275907672840 |
| ENSG00000167851 | CD300A | 18.22245426163390 |
| ENSG00000138646 | HERC5 | 18.1910050364818 |
| ENSG00000130584 | ZBTB46 | 18.15332666441260 |
| ENSG00000065665 | SEC61A2 | 18.0170351385369 |
| ENSG00000174953 | DHX36 | 17.910252210173200 |
| ENSG00000215533 | LINC00189 | 17.834309688777300 |
| ENSG00000272914 | None | 17.7196020355773 |
| ENSG00000130479 | MAP1S | 17.473070532293200 |
| ENSG00000168062 | BATF2 | 17.472625513114500 |
| ENSG00000118655 | DCLRE1B | 17.374287981320400 |
| ENSG00000116977 | LGALS8 | 17.304605609138900 |
| ENSG00000265681 | RPL17 | 17.125450720955800 |
| ENSG00000285572 | LOC124903183 | 16.978924845885500 |
| ENSG00000228782 | MRPL45P2 | 16.97147222801250 |
| ENSG00000279700 | None | 16.87923888075620 |
| ENSG00000205336 | ADGRG1 | 16.777870255173000 |
| ENSG00000099810 | MTAP | 16.08914083926290 |
| ENSG00000112701 | SENP6 | 16.06262207695550 |
| ENSG00000139567 | ACVRL1 | 15.763745636194800 |
| ENSG00000143498 | TAF1A | 15.610814872328800 |
| ENSG00000109189 | USP46 | 15.564885606515400 |
| ENSG00000285230 | RALY-AS1 | 15.30756668141710 |
| ENSG00000156603 | MED19 | 14.795305081645200 |
| ENSG00000243753 | HLA-L | 14.74276806288230 |
| ENSG00000116266 | STXBP3 | 14.44610113645830 |
| ENSG00000242372 | EIF6 | 14.306978713722000 |
| ENSG00000130475 | FCHO1 | 14.081378024154300 |
| ENSG00000172661 | WASHC2C | 14.03444450364880 |
| ENSG00000196914 | ARHGEF12 | 13.891338955663000 |
| ENSG00000167123 | CERCAM | 13.887461222319500 |
| ENSG00000120437 | ACAT2 | 13.423395981846200 |
| ENSG00000206561 | COLQ | 13.211683660193400 |
| ENSG00000158234 | FAIM | 12.795219888069900 |
| ENSG00000130164 | LDLR | 12.783517164443700 |
| ENSG00000238197 | PAXBP1-AS1 | 12.547185891521900 |
| ENSG00000168944 | CEP120 | 12.50272241097080 |
| ENSG00000204120 | GIGYF2 | 12.480600927302300 |
| ENSG00000104613 | INTS10 | 12.461893195845900 |
| ENSG00000187860 | CCDC157 | 12.306210053524400 |
| ENSG00000121297 | TSHZ3 | 12.303531016780300 |
| ENSG00000287185 | None | 11.97648662811860 |
| ENSG00000228590 | MIR4432HG | 11.26841293238390 |
| ENSG00000137500 | CCDC90B | 11.015845667252500 |
| ENSG00000205517 | RGL3 | 10.89307620045070 |
| ENSG00000107798 | LIPA | 10.8670918663434 |
| ENSG00000234694 | CDC20-DT | 10.832363297741200 |
| ENSG00000108433 | GOSR2 | 10.753068081769900 |
| ENSG00000143376 | SNX27 | 10.40973528727160 |
| ENSG00000277452 | RN7SL473P | 10.368597253529900 |
| ENSG00000021574 | SPAST | 10.041572189590900 |
| ENSG00000146247 | PHIP | 9.99350962132432 |
| ENSG00000162654 | GBP4 | 9.938286001162410 |
| ENSG00000164938 | TP53INP1 | 9.791344698122470 |
| ENSG00000099995 | SF3A1 | 9.50775834668787 |
| ENSG00000164096 | C4orf3 | 9.265639034934850 |
| ENSG00000014641 | MDH1 | 9.110312904561150 |
| ENSG00000005194 | CIAPIN1 | 9.028930191305240 |
| ENSG00000143126 | CELSR2 | 9.00563683328454 |
| ENSG00000164116 | GUCY1A1 | 8.510219882510530 |
| ENSG00000005108 | THSD7A | 8.291765856983990 |
| ENSG00000143740 | SNAP47 | 8.118651386199010 |
| ENSG00000179195 | ZNF664 | 7.958775489942390 |
| ENSG00000182405 | PGBD4 | 7.613144515042960 |
| ENSG00000204580 | DDR1 | 7.596690827306250 |
| ENSG00000101442 | ACTR5 | 7.308052894644130 |
| ENSG00000151465 | CDC123 | 7.105604796712320 |
| ENSG00000105287 | PRKD2 | 6.975229389578540 |
| ENSG00000177374 | HIC1 | 6.839335834598390 |
| ENSG00000149243 | KLHL35 | 6.746374740387400 |
| ENSG00000111424 | VDR | 6.508362365255180 |
| ENSG00000165801 | ARHGEF40 | 6.480624571226690 |
| ENSG00000183655 | KLHL25 | 6.161989356974760 |
| ENSG00000185022 | MAFF | 6.152623154817040 |
| ENSG00000225936 | SLC18A2-AS1 | 5.580913683169420 |
| ENSG00000172732 | MUS81 | 5.437408840035430 |
| ENSG00000104897 | SF3A2 | 5.307393652087350 |
| ENSG00000105889 | STEAP1B | 5.216554713811680 |
| ENSG00000130433 | CACNG6 | 5.068447864784460 |
| ENSG00000119403 | PHF19 | 5.021060459748110 |
| ENSG00000235652 | EPM2A-DT | 4.840062801140240 |
| ENSG00000237950 | LINC02918 | 4.469114504697220 |
| ENSG00000204592 | HLA-E | 4.255634230346330 |
| ENSG00000250508 | LINC02701 | 4.227607509435040 |
| ENSG00000272501 | None | 3.9961060732947800 |
| ENSG00000232855 | None | 3.936572967913540 |
| ENSG00000286230 | None | 3.6577890221471400 |
| ENSG00000078081 | LAMP3 | 3.5295682534998900 |
| ENSG00000065809 | FAM107B | 3.4824983222218200 |
| ENSG00000143436 | MRPL9 | 3.2917005528557200 |
| ENSG00000140564 | FURIN | 3.2607523889460200 |
| ENSG00000198933 | TBKBP1 | 3.2009556611566900 |
| ENSG00000250535 | STK19B | 3.1796770540662500 |
| ENSG00000165609 | NUDT5 | 2.861384935659670 |
| ENSG00000279670 | None | 2.464684143680830 |
| ENSG00000225265 | TAF1A-AS1 | 2.4522994405849100 |
| ENSG00000171385 | KCND3 | 2.115536770283540 |
| ENSG00000214212 | C19orf38 | 1.963936399365130 |
| ENSG00000198353 | HOXC4 | 1.9590255039129900 |
| ENSG00000187446 | CHP1 | 1.657305152735630 |

**Supplementary Table 3: Comparison of Clinical Characteristics and Risk Factors Across True Positive, False Positive, True Negative, and False Negative Groups.**

|  | **TP**  **n=88** | **FP**  **n=20** | **p value**  **(TP vs FP)** | **TN**  **n=80** | **FN**  **n=8** | **p value**  **(TN vs FN)** |
| --- | --- | --- | --- | --- | --- | --- |
| **Age, mean (SD)** | 61.5 (7.3) | 58.4 (10.5) | 0.1111 | 54.4 (7.2) | 53.5 (10.9) | 0.7602 |
| **Female, %** | 42% | 45.0% | 1 | 48.8% | 25.0% | 0.3616 |
| **Diabetes, %** | 12.5% | 15.0% | 1 | 11.2% | 12.5% | 1 |
| **Hypertension, %** | 52.3% | 30.0% | 0.1207 | 28.7% | 62.5% | 0.1196 |
| **High cholesterol, %** | 39.8% | 30.0% | 0.5770 | 21.2% | 0.0% | 0.3261 |
| **Esteatosis, %** | 33.0% | 20.0% | 0.386 | 26.2% | 25.0% | 1 |
| **Smoker, %** | 15.9% | 10.0% | 0.7468 | 13.8% | 25.0% | 0.7395 |
| **CAC, mean (SD)** | 228.0 (429.0) | 0.0 (0.0) | 0.0196 | 0.0 (0.0) | 30.3 (55.4) | <0.0001 |
| **Obesity, %** | 36.4% | 35.0% | 1.0 | 40.0% | 50.0% | 0.8639 |
| **Statins use** | 31.8% | 20.0% | 0.4391 | 13.8% | 25% | 0.7395 |

**Supplementary Table 4. Comparative discriminatory capacity between the ASCVD risk equation and the combined AI model.**

| **Risk equation** | | **Low Risk (n=125)** | | **Moderate Risk (n=41)** | | **High Risk (n=30)** | |
| --- | --- | --- | --- | --- | --- | --- | --- |
|  |  | CAC=0 | CAC>0 | CAC=0 | CAC>0 | CAC=0 | CAC>0 |
| **CT Scan (comparator)** | | 76 | 49 | 11 | 30 | 13 | 17 |
| **Combined AI Model classification** | **CAC =0** | 63 | 6 | 8 | 1 | 9 | 1 |
|  | **CAC >0** | 13 | 43 | 3 | 29 | 4 | 16 |

Supplementary References

1. Khananshvili D. The SLC8 gene family of sodium-calcium exchangers (NCX) - structure, function, and regulation in health and disease. Mol Aspects Med. 2013;34(2-3):220-35.

2. Liu A, Chen Z, Li X, et al. C5a-C5aR1 induces endoplasmic reticulum stress to accelerate vascular calcification via PERK-eIF2α-ATF4-CREB3L1 pathway. Cardiovasc Res. 2023;119(15):2563-2578.

3. Spoto B, Kakkar R, Lo L, et al. Serum Erythroferrone Levels Associated with Mortality and Cardiovascular Events in Hemodialysis and in CKD Patients: A Two Cohorts Study. J Clin Med. 2019;8(4):.

4. Cui F, Sun J, Mi H, et al. Chronic intermittent hypobaric hypoxia improves iron metabolism disorders via the IL-6/JAK2/STAT3 and Epo/STAT5/ERFE signalling pathways in metabolic syndrome rats. J Trace Elem Med Biol. 2023;79:127259.

5. Malhotra R, Mauer AC, Lino Cardenas CL, et al. HDAC9 is implicated in atherosclerotic aortic calcification and affects vascular smooth muscle cell phenotype. Nat Genet. 2019;51(11):1580-1587.

6. Duan S, Luo X, Dong C. Identification of susceptibility modules for coronary artery disease using a genome wide integrated network analysis. Gene. 2013;531(2):347-54.

7. Bonaventura A, Montecucco F, Dallegri F, et al. Novel findings in neutrophil biology and their impact on cardiovascular disease. Cardiovasc Res. 2019;115(8):1266-1285.

8. Pertiwi KR, van der Wal AC, Pabittei DR, et al. Neutrophil Extracellular Traps Participate in All Different Types of Thrombotic and Hemorrhagic Complications of Coronary Atherosclerosis. Thromb Haemost. 2018;118(6):1078-1087.

9. Zaric BL, Radovanovic JN, Gluvic Z, et al. Atherosclerosis Linked to Aberrant Amino Acid Metabolism and Immunosuppressive Amino Acid Catabolizing Enzymes. Front Immunol. 2020;11:551758.

10. Ren Y, Li Z, Li W, et al. Arginase: Biological and Therapeutic Implications in Diabetes Mellitus and Its Complications. Oxid Med Cell Longev. 2022;2022:2419412.

11. Baccarelli AA, Byun HM. Platelet mitochondrial DNA methylation: a potential new marker of cardiovascular disease. Clin Epigenetics. 2015;7(1):44.

12. Holvoet P, Vanhaverbeke M, Bloch K, et al. Low MT-CO1 in Monocytes and Microvesicles Is Associated With Outcome in Patients With Coronary Artery Disease. J Am Heart Assoc. 2016;5(12):.

13. Rönn T, Volkov P, Gillberg L, et al. Impact of age, BMI and HbA1c levels on the genome-wide DNA methylation and mRNA expression patterns in human adipose tissue and identification of epigenetic biomarkers in blood. Hum Mol Genet. 2015;24(13):3792-813.
