# Supplementary figures and images for: Liquid biopsy based on whole blood transcriptome and artificial intelligence for the prediction of coronary artery calcification: a pilot study
Source: Eur Heart J Digit Health. 2025 May 2;6(4):587–94. doi: 10.1093/ehjdh/ztaf042 (PMC12282340; doi:10.1093/ehjdh/ztaf042)

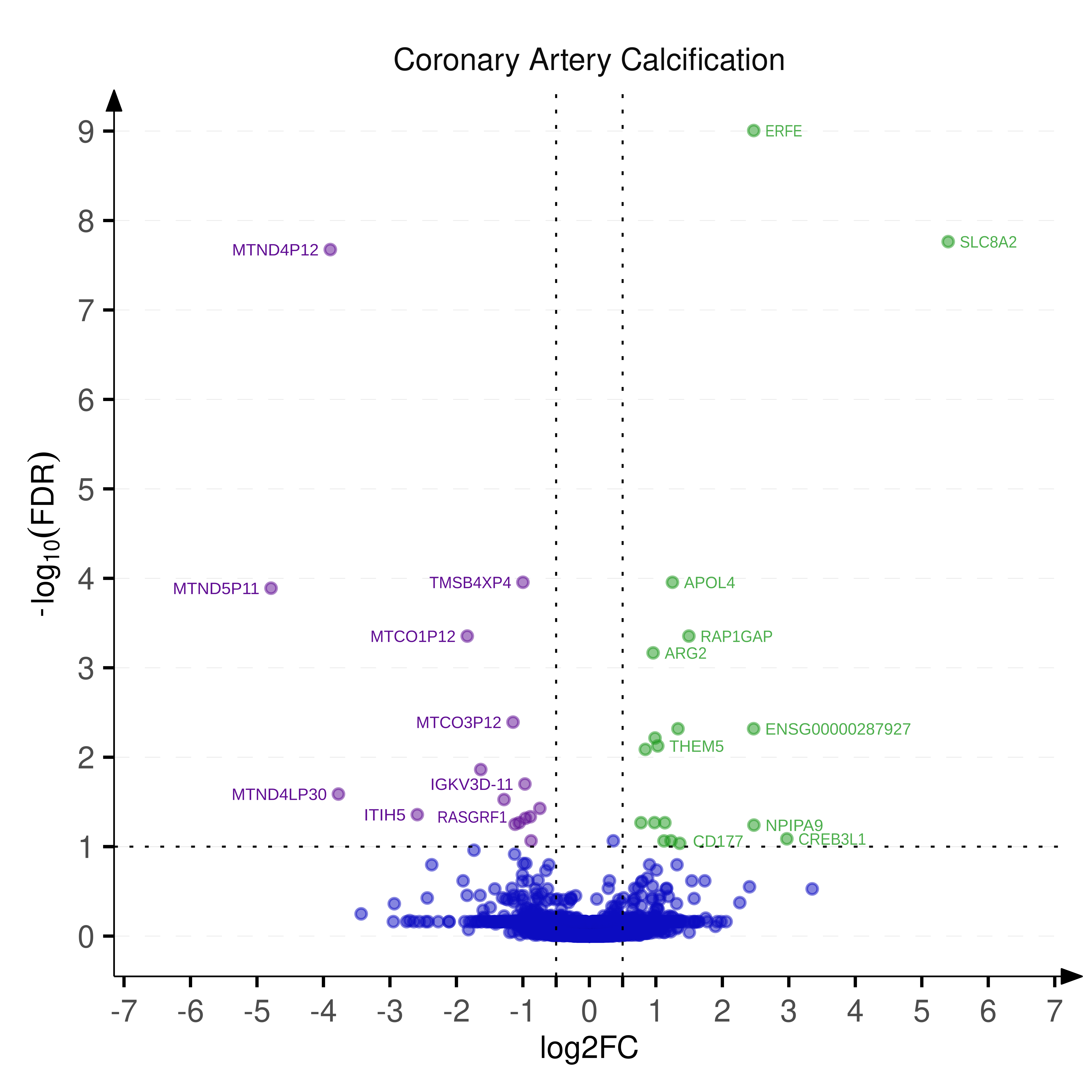

Supplement: ztaf042_Supplementary_Data [file ztaf042_supplementary_data.zip › Supplementary Figure 1.png]
